# miRNAs Flowing Up and Down: The Concerto of Psoriasis

**DOI:** 10.3389/fmed.2021.646796

**Published:** 2021-02-26

**Authors:** Yang Xiuli, Wang Honglin

**Affiliations:** Key Laboratory of Cell Differentiation and Apoptosis of Chinese Ministry of Education, Translational Medicine Center, Shanghai Institute of Immunology, Shanghai General Hospital, Shanghai Jiao Tong University School of Medicine, Shanghai, China

**Keywords:** psoriasis, miRNAs, epidermal hyperplasia, keratinocytes, CD4^+^ T cells

## Abstract

Psoriasis is a chronic immune-mediated skin disease, whose hallmarks include keratinocyte hyperproliferation and CD4^+^ T cell subsets imbalance. Dysregulated microRNAs (miRNAs) identified in psoriasis have been shown to affect keratinocyte and T cell functions, with studies on the molecular mechanisms and intrinsic relationships of the miRNAs on the way. Here, we focus on the dysregulated miRNAs that contribute to the two hallmarks of psoriasis with the miRNA target genes confirmed. We review a network, in which, upregulated miR-31/miR-203/miR-155/miR-21 and downregulated miR-99a/miR-125b facilitate the excessive proliferation and abnormal differentiation of psoriatic keratinocytes; upregulated miR-210 and downregulated miR-138 work in concert to distort CD4^+^ T cell subsets balance in psoriasis. The miRNAs exert their functions through regulating key psoriasis-associated transcription factors including NF-κB and STAT3. Whether flowing up or down, these miRNAs collaborate to promote the development and maintenance of psoriasis.

## Introduction

Psoriasis is a chronic immune-mediated skin disease driven by the coordination of genetic susceptibility, environmental triggers, and dysfunctional immune system, which is featured by the abnormal interplay between keratinocytes and T cells. Approximately 125 million people worldwide suffer from psoriasis, which substantially impacts their quality of life. Psoriasis vulgaris, as the commonest type of psoriasis, appears as well-demarcated, erythematous, scaly plaques, and accounts for about 80–90% of cases clinically (1, 2). To date, psoriasis does not have a cure, and the current therapies are aimed to achieve symptomatic relief. Thus, the pathogenic mechanisms of psoriasis have been intensively studied to benefit clinical practice.

Initially characterized by excessive proliferation and aberrant differentiation of epidermal keratinocytes, psoriasis was then found to involve T cell dysfunction, which led to the recognization of a dysregulated interplay between keratinocytes and infiltrating immune cells. Herein, interleukins (ILs) (IL-1, IL-17, IL-22, IL-23, etc.), tumor necrosis factor-α (TNF-α), interferon-γ (IFN-γ), and transforming growth factor-β (TGF-β) are key players for cell-cell crosstalks (3–5). As for the non-protein-coding regulatory elements, altered miRNA expression profiles were uncovered in psoriatic skin, blood, and hair samples (6, 7), which profoundly influences our knowledge of the driving force and regulatory counterpart in psoriasis.

miRNAs are a class of non-coding RNA molecules with ~22 nucleotides in length (8). Once loaded into the RNA-induced silencing complex (RISC), miRNAs recognize and bind to the complementary sequences in the 3′ untranslated region (3′UTR) of target mRNAs, leading to translational repression or degradation of the target mRNAs (9). Since the altered expression of miRNAs in psoriasis was first described in 2007 (10), more than 250 miRNAs have been identified to be differentially expressed in psoriatic skin or blood (7). miRNAs hold the potentials to regulate the proliferation, differentiation, apoptosis, and cytokine production of keratinocytes, as well as the activation and effection of T cell subsets (11–14). Additionally, circulating miRNAs detected in the sera of psoriasis patients showed correlations with Psoriasis Area Severity Index (PASI) scores and may serve as promising biomarkers for diagnosis, disease progression, and therapy evaluation (15, 16). Moreover, genetic polymorphisms of miRNAs are associated with psoriasis susceptibility (17). Therefore, a layer of regulatory mechanisms mediated by miRNAs provides new avenues to reveal psoriasis pathogenesis, thus facilitating the invention of advanced therapeutic strategies for psoriasis. Given that a handful of miRNAs have been confirmed with functional target mRNAs in psoriasis, we focus on the functions and signaling mechanisms of miR-31, miR-203, miR-155, miR-21, miR-125b, miR-99a, miR-146a, miR-210, and miR-138 in this review.

## miRNAs Regulatory Networks in Psoriatic Keratinocytes

Hallmarks of psoriasis include keratinocyte hyperproliferation and abnormal differentiation, accompanied by the aberrant production of inflammatory cytokines and chemokines from keratinocytes (5). By reviewing recent literature, we find that among the differentially expressed miRNAs in psoriasis, several miRNAs form a complementary regulatory network to impair the balance between proliferation and differentiation of keratinocytes. Specifically, upregulated miR-31/miR-203/miR-155/miR-21 and downregulated miR-125b/miR-99a work in concert to facilitate epidermal hyperplasia by targeting relevant mRNAs (e.g., ppp6c, FIH-1, p63, PTEN, AKT3, FGFR2, and IGF-1R) in psoriatic keratinocytes.

### Upregulated miRNAs in Psoriatic Keratinocytes

#### miR-31

miR-31 is one of the most overexpressed miRNAs in psoriatic keratinocytes (14). Protein phosphatase 6 (ppp6c) and factor-inhibiting hypoxia-inducible factor 1 (FIH-1) have been reported as the target genes of miR-31 in psoriasis (14, 18). Ppp6c, a negative regulator that restricts the G1 to S phase progression, is targeted by miR-31 in human psoriatic epidermis, thus contributing to the basal keratinocyte proliferation and epidermal hyperplasia (14). Knock out of FIH-1, a negative regulator that mediates Notch hydroxylation, enhances keratinocyte differentiation through Notch activation. Thus, overexpressed miR-31 leads to keratinocyte differentiation, in part, via inhibiting the expression of FIH-1 proteins in human epidermal keratinocytes (HEKs) and human corneal epithelial keratinocytes (HCEKs) (18). In addition, Borska et al. (19) identified a significant negative relationship between Endothelin-1 (ET-1) and miR-31 in psoriasis patients. According to their results, miR-31 may modulate apoptosis of psoriatic keratinocytes via inhibiting ET-1, which is produced by psoriatic keratinocytes to suppress keratinocyte apoptosis. Collectively, miR-31 functions as a multi-functional miRNA which plays pro-proliferative and pro-differentiative roles and modulates apoptosis in psoriatic keratinocytes. Notably, it has been demonstrated that FIH-1 is markedly increased in psoriastic keratinocytes, which bear responsibility for the seemingly contradictory functions of miR-31. It's worth exploring whether some unknown mechanisms exist to counteract the inhibitory effect of miR-31 on FIH-1.

#### miR-203

miR-203 has been identified as a keratinocyte-derived miRNA and its upregulation plays a critical role in the pathogenesis of psoriasis (10, 20). Owing to its ability to directly target and negatively regulate several epidermal genes, miR-203 participates in the balance of keratinocyte proliferation and differentiation. As a member of the p53 family, protein 63 (p63) functions as a determinant for the fate of keratinocytes (21, 22). Via binding to the 3′ UTR of p63 mRNA, miR-203 blocks the cell cycle at the G0/G1 phase and promotes the differentiation of keratinocytes (23, 24). Suppressor of cytokine signaling 3 (SOCS-3), the first reported target gene of miR-203, has also been identified to regulate keratinocyte proliferation and differentiation (10). Moreover, a recent study (25) has shown that liver X receptor-α (LXR-α) and peroxisome proliferator-activated receptor-γ (PPAR-γ) are remarkably downregulated in psoriatic lesions, and the overexpression of each gene is sufficient to inhibit keratinocyte proliferation. miR-203 negatively regulates the expression of LXR-α/PPAR-γ by directly targeting 3′ UTRs of their mRNAs, suggesting that the miR-203-LXR-α /PPAR-γ axis is crucially involved in the hyperproliferative phenotype of psoriatic keratinocytes and may provide drug targets for psoriasis treatment.

#### miR-155

miR-155 is significantly increased in skin lesions and peripheral blood mononuclear cells (PBMCs) of psoriasis patients and plays indispensable roles in keratinocyte proliferation, apoptosis, and inflammatory responses (26–28). A previous study (28) demonstrated that miR-155 could promote keratinocyte proliferation and inhibit apoptosis through the phosphatase and tension homolog deleted on chromosome 10 (PTEN) signaling pathway in psoriasis. PTEN is downregulated in psoriasis and loss of PTEN may cause an accumulation of phosphatidylinositol-3, 4, 5-trisphosphate (PIP3), which further increases AKT activity and leads to decreased apoptosis and increased proliferation (29, 30). Moreover, knockdown of miR-155 in HaCaT cells significantly increased cells in the G0/G1 phase and decreased those in the G2/M phase. Further results indicated that miR-155 might regulate p27 rather than p21 in HaCaT cells to promote G1 to S phase transition (28). Taken together, miR-155 plays multiple roles in keratinocytes, including cell proliferation and apoptosis, providing a potential therapeutic choice for treating psoriasis (26–28).

#### miR-21

miR-21 is significantly upregulated in psoriatic skin lesions, dermal T cells, and blood samples (10, 31). This miRNA can regulate the proliferation and apoptosis of psoriatic keratinocytes by binding to the 3′ UTR of caspase-8 mRNA (32, 33). The lncRNA maternally expressed gene 3 (MEG3), a competing endogenous RNA of miR-21, is significantly downregulated in TNF-α-stimulated HaCaT cells and psoriatic skin samples. MEG3 inhibits proliferation and promotes apoptosis of activated HaCaT by targeting miR-21, which further inhibits the expression of caspase-8. Thus, the MEG3/miR-21/caspase-8 axis regulates the proliferation and apoptosis of psoriatic keratinocytes, highlighting miR-21 as a promising drug target for the treatment of psoriasis (32).

### Downregulated miRNAs in Psoriatic Keratinocytes

#### miR-125b

miR-125b is one of the most downregulated miRNAs in psoriatic keratinocytes (10, 34, 35). A previous study (35) demonstrated that fibroblast growth factor receptor 2 (FGFR2), a receptor upregulated in psoriatic keratinocytes (36), was a target gene of miR-125b. Via base-pairing with the 3′ UTR of FGFR2 mRNA to suppress its expression, miR-125b inhibited the proliferation of primary human keratinocytes. miR-125b can also inhibit the expression of the upstream protein BRD4 and the ligand Jagged-1 of the Notch signaling pathway, thereby inhibiting keratinocyte proliferation in psoriasis via activating the BRD4/Notch signaling pathway (34). AKT3 is another target gene of miR-125b. Overexpression of miR-125b blocks the AKT pathway and inhibits the proliferation of HEKs (37). Moreover, miR-125b targets Ubiquitin-specific peptidase 2 (USP2), whose ablation in keratinocytes reduces proliferation rate and increases differentiation (38). According to these observations, it is reasonable to conclude that miR-125b acts as a preventive factor for psoriasis through inhibiting proliferation and enhancing differentiation of keratinocytes.

#### miR-99a

miR-99a expression is downregulated in PBMCs and skin lesions of psoriasis patients compared with healthy controls (16, 39, 40). Insulin-like growth factor 1 receptor (IGF-1R) is a direct target of miR-99a, as demonstrated by a reporter assay. miR-99 decreases the protein levels of IGF-1R, which consequently inhibits keratinocyte proliferation and promotes keratinocytes toward terminal differentiation. IGF-1R signaling upregulates the expression of miR-99a, which in turn downregulates the expression of IGF-1R as negative feedback (39). Apart from IGF-1R, frizzled-5 (FZD5) and frizzled-8 (FZD8) are both confirmed targets of miR-99a in psoriasis. Shen et al. (40) showed that miR-99a inhibited keratinocyte proliferation by targeting FZD5/FZD8, which affects downstream factors β-catenin and cyclinD1. Taken together, miR-99a inhibits keratinocyte proliferation while promotes terminal differentiation via targeting IGF-1R and FZD5/8. Such regulatory mechanisms are impaired in psoriatic skin due to the deficiency of miR-99a.

**Figure 1:**
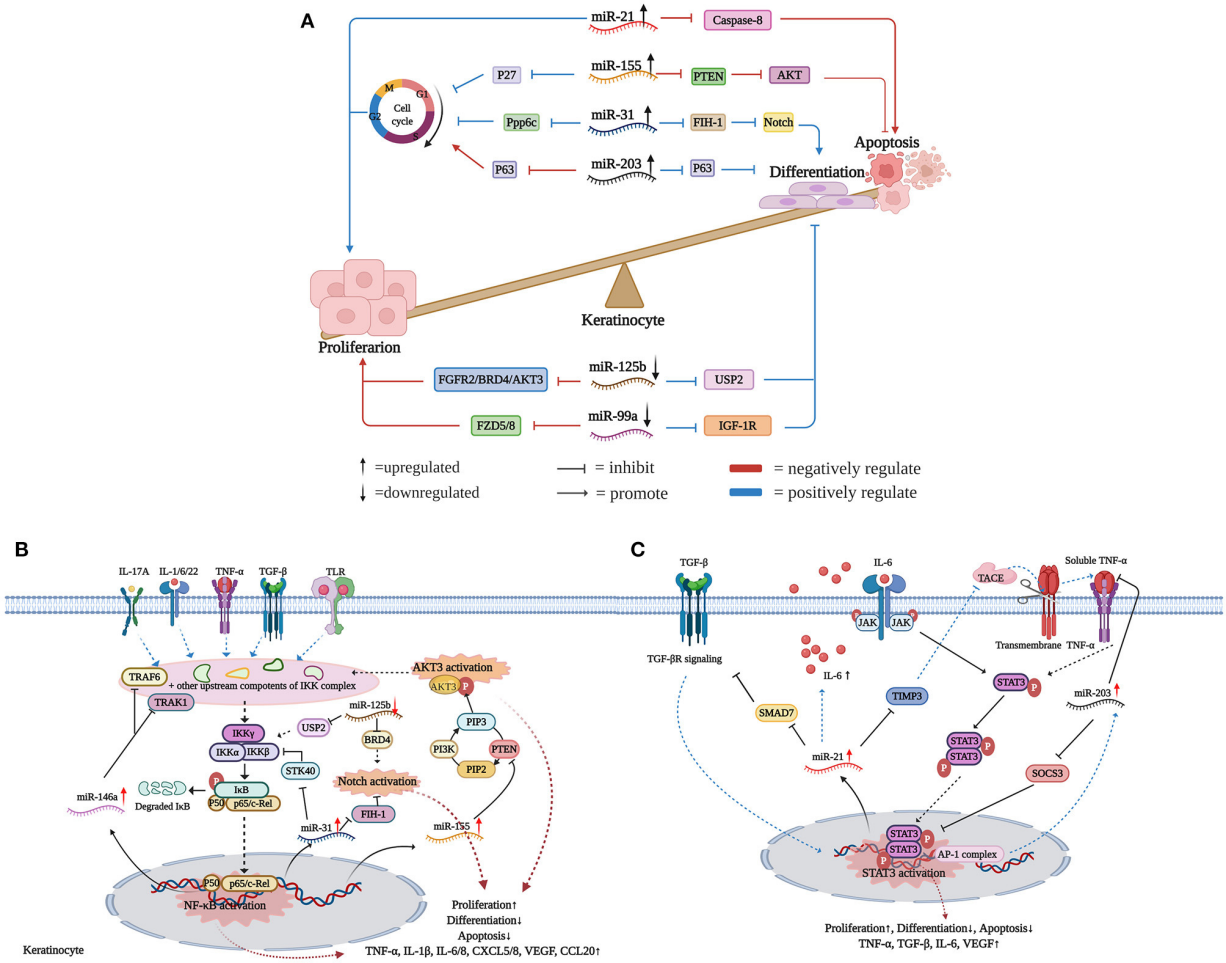
miRNAs flowing up and down to facilitate keratinocyte hyperproliferation. **(A)** Upregulated miR-31/miR-203/miR-155/miR-21 and downregulated miR-99a/miR-125b work in concert to promote psoriasis epidermal hyperplasia. Actually, whether flowing up or down, these dysregulated miRNAs are interconnected, interactional, and complementary in the development of psoriatic keratinocyte hyperproliferation. **(B)** miRNAs regulatory network in the NF-κB/Notch/AKT signaling pathways. **(C)** miRNAs regulatory network in the STAT3 signaling pathway. Created with Biorender.com.

As summarized in Figure 1A, we find that upregulated miR-31/miR-203/miR-155/miR-21 and downregulated miR-99a/miR-125b work in concert to promote the hyperproliferation and abnormal differentiation of psoriatic keratinocytes. The miRNA regulatory network is like “Yin” and “Yang,” which means whether flowing up or down, these dysregulated miRNAs are interconnected, interactional, and complementary in driving and maintaining psoriatic skin lesions. Promisingly, other miRNAs are likely to be added into this network with confirmed mRNA targets and established biological functions in the future. Psoriasis is a complex multifactorial disease without a cure, which should be attributed to the ill-defined pathogenic mechanisms. Undoubtedly, investigation of the mechanisms of epidermal hyperplasia and abnormal differentiation regulated by miRNAs is conducive to fully unveiling the pathogenesis of psoriasis.

### miRNAs Act in NF-κB/NOTCH/AKT Signaling Pathways

Classically, NF-κB dimers are sequestered in the cytoplasm through forming complexes with the inhibitory protein IκB (41). Once inflammatory mediators like TGF-β1, IL-1α, IL-6, IL-17A, TNF-α, IFN-γ, and IL-22 bind to their receptors, NF-κB is rapidly activated and translocated into the nuclei to promote the expression of target genes in keratinocytes (42). A few previous studies have demonstrated that miRNAs play vital roles in the NF-κB signaling pathway in psoriasis, acting either as inhibitors or activators (14, 43, 44). Increased miR-146a has a strong positive correlation with IL-17A in psoriasis patients and can be induced by IL-1β, TNF-α, and IL-17A in human proliferating keratinocytes, indicating its potential roles downstream of IL-17A/TNF-α signals (45). Via targeting TNF receptor-associated factor 6 (TRAF6) and IL-1 receptor-associated kinase 1 (IRAK1) mRNAs, overexpressed miR-146a inhibited the expression of IL-6, IL-8, TNF-α, C-C Motif Chemokine Ligand 20 (CCL20), and suppressed neutrophil chemoattraction by keratinocytes (17, 43, 46). However, miR-146a also targets atypical chemokine receptor 2 (ACKR2) in keratinocytes, resulting in enhanced inflammatory chemokine activity and increased infiltration of T cells into the psoriatic epidermis (47). Thus, whether miR-146a acts as a protective factor or a disease promoter in psoriasis is not fully clarified. Moreover, inflammatory mediators (e.g., TGF-β1, IL-1α, IL-6, IL-17A, TNF-α, IFN-γ, and IL-22) can directly or indirectly activate the NF-κB signaling pathway, which drives the transcription of miR-31 through increased promoter activity (14, 18, 44). In turn, miR-31 directly targets Serine/Threonine Kinase 40 (STK40), an inhibitor of NF-κB signals, thus maintaining the stable NF-κB activation through a positive feedback loop (44). Interestingly, STK40 is also targeted by miRNA-130a, resulting in the promotion of viability and inhibition of apoptosis in HaCaT cells (48). Through regulating NF-κB activation, upregulated miR-31 contributes to skin inflammation in psoriatic lesions by increasing the production of inflammatory mediators and leukocyte chemoattractants (e.g., IL-1β, IL-6, IL-8, CXCL1, CXCL5, CXCL8, and TNF-α) (14, 44).

Intriguingly, several miRNAs act as communicators between NF-κB and Notch signaling pathways. Firstly, Notch undergoes hydroxylation by FIH-1, while NF-κB-induced miR-31 is an endogenous negative regulator of FIH-1 expression (18). Secondly, through inhibiting the expression of USP2, miR-125b negatively regulates NF-κB activation in psoriasis. Thus, overexpression of miR-125b contributes to the decrease of NF-κB-dependent cytokine production and skin inflammation (38). Meanwhile, miR-125b also targets BRD4, a negative regulator of the Notch signaling pathway (34). Thirdly, p63 plays an important role in NF-κB and Notch signaling pathways via mediating the activation of the IKK and Jagged-1 complex, respectively. Previous studies have shown p63 is a common target of miR-125b and miR-203 in epidermal keratinocytes (21, 23, 49). All of these observations suggest that there is a close interplay between NF-κB/Notch signals and miRNAs.

PTEN-PI3K-AKT signaling is also aberrantly activated in psoriatic keratinocytes, and several miRNAs take part in this process. Among the three isoforms of AKT, only AKT3 is upregulated in psoriasis (37, 50). Correspondingly, miR-125b-5p, miR-181b-5p, and miR-320b have been demonstrated to target AKT3 mRNA, thus inhibiting the proliferation of HEKs (37, 51). Furthermore, cytokines like IL-1β, IFN-γ, and TNF-α bind to their receptors and trigger NF-κB activation, thereby increasing the transcription of miR-155 (28, 52, 53). Reciprocally, through targeting PTEN, a phosphatase that dephosphorylates PIP3 produced by PI3K, miR-155 indirectly enhances NF-κB activation (28). Similarly, another study found that miR-223 inhibits PTEN expression through binding to the 3′UTR of its mRNA, thereby increasing proliferation and inhibiting apoptosis of IL-22-stimulated keratinocytes (54). To some extent, these studies indicate that the PTEN-PI3K-AKT pathway is regulated by miRNAs in psoriasis (Figure 1B).

### miRNAs Act in Stat3 Signaling Pathway

miR-21 is significantly upregulated in psoriatic skin lesions and dermal T cells (12, 31, 55). There are three molecular mechanisms to induce miR-21 expression in psoriatic keratinocytes. Firstly, TGF-β is upregulated in psoriatic keratinocytes and triggers the transcription of miR-21 (31). Mothers against decapentaplegic homolog 7 (SMAD7), which inhibits TGF-βR expression through promoting its degradation, is a target of miR-21 in keratinocytes. Thus, TGF-β-induced miR-21 maintains its transcription by inhibiting SMAD7 expression (56, 57). Secondly, tissue inhibitor of matrix metalloproteinase 3 (TIMP-3), an inhibitor of TNF-α-converting enzyme (TACE), is downregulated in psoriatic keratinocytes. miR-21 inhibits epidermal TIMP-3 expression, which leads to enhanced release of soluble TNF-α from keratinocytes to promote skin inflammation (31, 58). Notably, increased miR-221 and miR-222 also target TIMP-3 in psoriatic skin lesions, thus underscoring the potential importance of TIMP-3 and matrix metalloproteases in the immunopathogenesis of psoriasis (55). Besides, through binding to the TNF-α receptor, the soluble form of TNF-α triggers STAT3 phosphorylation, which leads to translocation of STAT3 dimers into nuclei and increases miR-21 transcription. Thirdly, the impaired transcriptional activity of Jun/ activating protein 1 (AP-1) leads to an increase of IL-6 in JunB/c-Jun deficient mouse keratinocytes. Increased IL-6, in turn, binds to JAK receptors and further activates the transcriptional inducer STAT3, thus increasing the expression of miR-21 (31).

miR-203 upregulation is required for human keratinocyte differentiation. The upregulation of JunB and deregulation of c-Jun, contribute to increased miR-203 expression in keratinocytes (20). Besides, TNF-α-induced miR-203 in turn inhibits TNF-α via targeting its mRNA (59). Besides, Xu et al. (60) showed that miR-203 expression in keratinocytes is upregulated upon IL-17 stimulation, and miR-203 is a positive regulator of IL-17-induced vascular endothelial growth factor (VEGF) secretion. Increased miR-203 regulates keratinocyte functions through post-transcriptional suppression of its target mRNAs, including SOCS-3, p63, LXR-α, PPAR-γ, TNF-α, IL-24, and yet-to-be-defined targets. Notably, SOCS3 is a negative regulator of the STAT3 signaling pathway. Increased expression of miR203 leads to decreased SOCS3 in psoriatic skin, which consequently results in sustained activation of STAT3 signaling (10).

Apart from miR-21 and miR-203, several miRNAs have also been identified to regulate STAT3 signals. *In vitro* settings, the overexpression of hsa-miR-4516 downregulated STAT3 and induced human keratinocyte apoptosis by directly binding to STAT3 mRNA (61, 62). Even so, our knowledge about the interplay between miRNAs and STAT3 signals is limited (Figure 1C).

## miRNAs Work in Concert to Distort CD4^+^T Cell Subsets Balance

Although the pathogenesis of psoriasis remains elusive, the imbalance of CD4^+^ T cell subsets has been demonstrated to be a critical pathogenic factor, which involves Th1 and Th17 cell expansion and Treg cell dysfunction (4, 63, 64). Up to date, a series of miRNAs that regulate T cell fates and behaviors have been reported, while only a few of them have confirmed mRNA targets or established biological functions in CD4^+^ T cell subsets. Here we summarized the accessible knowledge of miR-210, miR-138, miR-21, miR-200a, and miR-146a that regulate CD4^+^ T cells in psoriasis.

The expression of miR-210 is increased in CD4^+^ T cells from patients with psoriasis and psoriasis mouse models. Mechanistically, TGF-β/IL-23 induces hypoxia-inducible factor-1α (HIF-1α), which recruits the histone acetyltransferase P300 to the miR-210 promoter region, thereby enhancing miR-210 expression in CD4^+^ T cells from psoriasis patients (13, 65). Moreover, the transcription factor forkhead box P3 (FOXP3), which is required for Treg cell development and function, is a target gene of miR-210. According to Zhao et al. (66), inhibition of miR-210 leads to increased FOXP3 and recovery of the immunosuppressive functions of Treg cells from patients with psoriasis. Meanwhile, miR-210 also promotes Th1/Th17 cell differentiation but precludes Th2 differentiation through repressing STAT6 and LYN expression via binding the 3′UTRs of their mRNAs. Besides, miR-210 overexpression leads to abnormal cytokine expression, namely increased IFN-γ/IL-17 and decreased IL-10/TGF-β production, in CD4^+^ T cells from patients with psoriasis. Notably, miR-210 is upregulated in keratinocytes, and overexpression of this miRNA increases the proliferation and chemokine secretion of keratinocytes, which recruit activated T cells into skin lesions (13). Taken together, increased miR-210 in CD4^+^ T cells and keratinocytes contribute to the formation of psoriatic skin lesions through several lines of directions. Intriguingly, a recent study developed nanocarrier gel containing miR-210 antisense (NG-anti-miR-210) to topically inhibit miR-210 expression in the imiquimod-induced mouse model of psoriasis. The results showed that inhibition of miR-210 significantly alleviates psoriasis-like inflammation in mice with a decreased proportion of Th1 and Th17 cells in dermal and splenic cells, which paves the way for RNA drug development for psoriasis (67).

**Figure 2:**
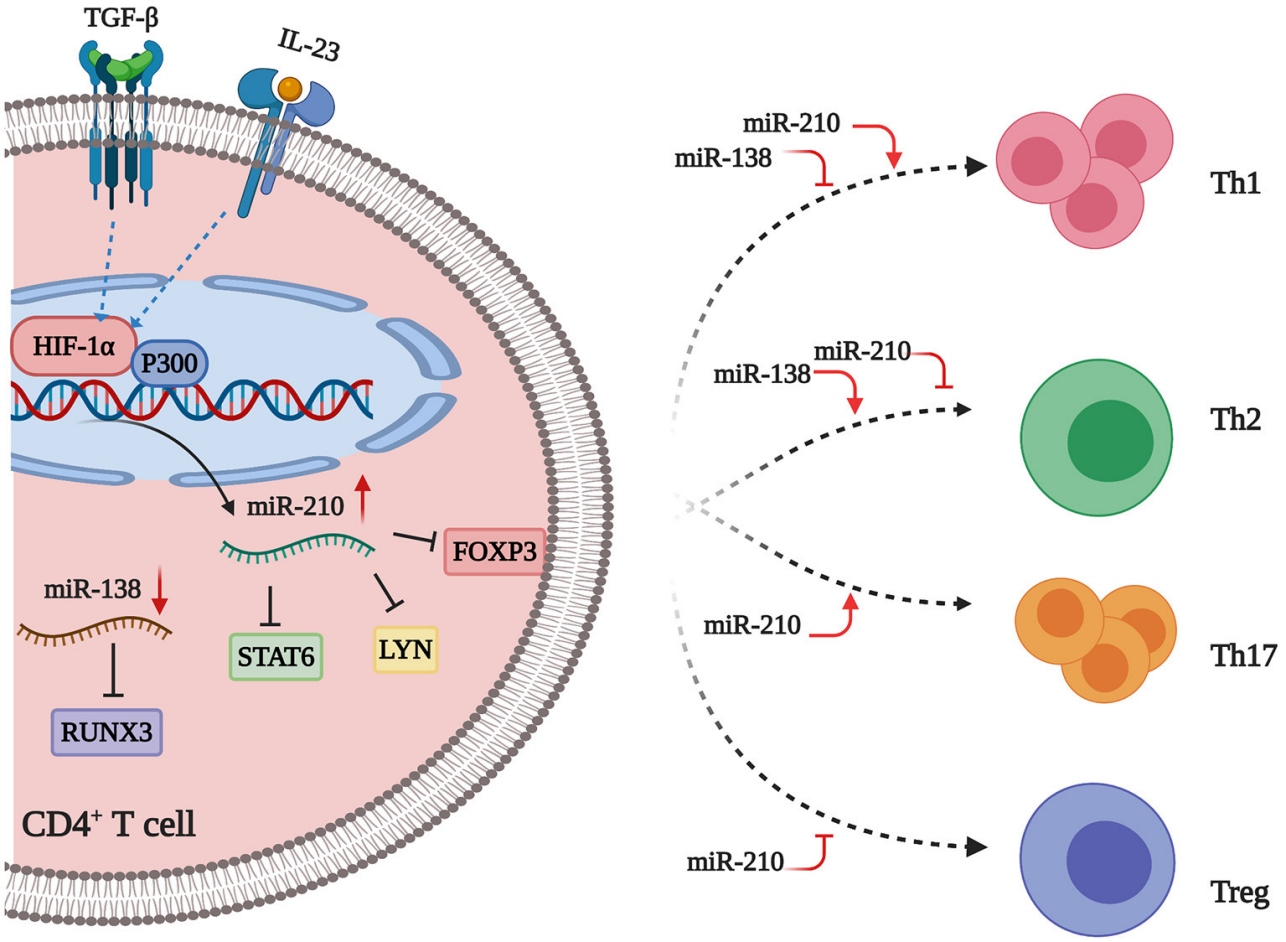
miRNAs flowing up and down to distort CD4^+^ T cell subsets balance. miR-210 is increased in psoriasis. Inflammatory factors (e.g., TGF-β and IL-23) stimulate the expression of HIF-1α, which can recruit P300 to the miR-210 promoter region, thus resulting in the upregulation of miR-210 expression. This miRNA directly targets FOXP3, which is a master transcription factor for the differentiation of Treg cells, thus leading to a decreased number of Treg cells. Besides, miR-210 can also repress STAT6 and LYN expression by binding to the 3′ UTRs of their mRNAs, thereby promoting Th1/Th17 cell differentiation and inhibiting Th2 cell differentiation in CD4^+^ T cells. MiR-138 is decreased in psoriatic lesions. This miRNA suppresses RUNX3 expression through binding to the 3′UTR of RUNX3 mRNA. Thus, downregulated miR-138 leads to the upregulation of RUNX3, which increases the Th1/Th2 ratio in CD4^+^ T cells. Taken together, upregulated miR-210 and downregulated miR-138 work in concert to distort CD4^+^ T cell subsets balance in psoriasis. Created with Biorender.com.

miR-138 is significantly downregulated in CD4^+^ T cells from psoriasis patients (11, 55). Moreover, transfection with miR-138 mimics into CD4^+^ T cells from psoriasis patients results in inhibition of Runt-related transcription factor 3 (RUNX3) via binding to the 3′UTR of its mRNA, which then decreases the ratio of Th1/Th2 (11). Notably, RUNX3, as a psoriasis susceptibility gene, plays an important role in the differentiation of T cells in psoriasis, through both modulating the balance of Th1/Th2 and regulating the differentiation of Th17 and Th22 cells (68). Therefore, miR-138 may have protective effects against T-cell-mediated auto-inflammation via inhibiting RUNX3 expression. Additionally, upregulated miR-21 suppresses the apoptosis of activated T cells in psoriasis. Furthermore, knock-down of miR-21 in activated human primary CD4^+^ T cells results in increased apoptosis, suggesting that miR-21 may contribute to the persistence of CD4^+^ T cell infiltration in psoriatic skin (12). Upregulated miR-200a in CD4^+^ T cells induce immune dysfunction through increasing Th17/Treg ratio and pro-inflammatory cytokine production in psoriasis patients (69). In contrast, miR-146a is preferentially expressed by Treg cells, highlighting its immunomodulatory roles in psoriatic skin (10, 70). Upregulated miR-210 and downregulated miR-138 work in concert to distort CD4^+^ T cell subsets balance (Figure 2). The specific functions and related mechanisms of other dysregulated miRNAs in psoriatic T cells need to be investigated.

## Conclusion

In this review, we focus on miRNAs with identified target genes and established biological functions in psoriatic keratinocytes and CD4^+^ T cell subsets. Herein, we find that upregulated miR-31/miR-203/miR-155/miR-21 and downregulated miR-99a/miR-125b contribute to the hyperproliferation and abnormal differentiation of psoriatic keratinocytes through synergistic networks. The mechanism of this miRNA regulatory network is like “Yin” and “Yang” in Chinese medicine, which means whether flowing up or down, these dysregulated miRNAs are interconnected, interactional, and complementary to promote keratinocyte hyperproliferation and abnormal differentiation. Promisingly, other miRNAs are likely to be added into this network with confirmed mRNA targets and established biological functions in the future. A parallel miRNA network exists in CD4^+^ T cell subsets from psoriasis patients. Upregulated miR-210 and downregulated miR-138 work in concert to distort CD4^+^ T cell subsets balance, which is a crucial pathomechanism in psoriasis. Notably, many miRNAs function through NF-κB, Notch, PTEN-PI3K-AKT3, and STAT3 signaling pathways. Undoubtedly, further investigation of miRNA-associated regulatory mechanisms involved in psoriasis is conducive to the invention of new strategies for treating this refractory disease. We present a novel perspective of miRNAs in psoriasis, by viewing them not as passive participators but as active collaborators.

## Author Contributions

WH contributed to the conception of the review article. YX contributed to the manuscript preparation. Both authors contributed to the article and approved the submitted version.

## Conflict of Interest

The authors declare that the research was conducted in the absence of any commercial or financial relationships that could be construed as a potential conflict of interest.
